# Identification of circulating miRNAs differentially expressed in patients with Limb-girdle, Duchenne or facioscapulohumeral muscular dystrophies

**DOI:** 10.1186/s13023-022-02603-3

**Published:** 2022-12-27

**Authors:** José Luis García-Giménez, Elena R. García-Trevijano, Ana I. Avilés-Alía, José Santiago Ibañez-Cabellos, Miquel Bovea-Marco, Teresa Bas, Federico V. Pallardó, Juan R. Viña, Rosa Zaragozá

**Affiliations:** 1 Center for Biomedical Network Research On Rare Diseases (CIBERER), Institute of Health Carlos III, Valencia, Spain; 2 INCLIVA Health Research Institute, Valencia, Spain; 3 Department of Physiology, Faculty of Medicine and Dentistry, University of Valencia, Valencia, Spain; 4 EpiDisease S.L. (Spin-Off CIBERER), Valencia, Spain; 5 Department of Biochemistry and Molecular Biology, Faculty of Medicine and Dentistry, University of Valencia, Valencia, Spain; 6 Institute for Health Research La Fe, IISLaFe, Valencia, Spain; 7 Spine Surgery Unit, Hospital Universitari i Politècnic La Fe, Valencia, Spain; 8 Department of Human Anatomy and Embryology, Faculty of Medicine and Dentistry, University of Valencia, Avda. Blasco Ibañez 15, 46010 Valencia, Spain

**Keywords:** Limb girdle muscular dystrophies, Circulating miRs, Molecular signature, Duchenne muscular dystrophy, Facioscapulohumeral muscular dystrophy

## Abstract

**Background:**

Limb-girdle muscular dystrophy (LGMD) is a rare neuromuscular disease including a growing and heterogeneous number of subtypes with variable phenotype. Their clinical and histopathological characteristics frequently overlap with other neuromuscular dystrophies. Our goal was to identify, by a non-invasive method, a molecular signature including biochemical and epigenetic parameters with potential value for patient prognosis and stratification.

**Results:**

Circulating miRNome was obtained by smallRNA-seq in plasma from LGMD patients (n = 6) and matched-controls (n = 6). Data, validated by qPCR in LGMD samples, were also examined in other common muscular dystrophies: Duchenne (DMD) (n = 5) and facioscapulohumeral muscular dystrophy (FSHD) (n = 4). Additionally, biochemical and clinical parameters were analyzed. miRNome analysis showed that thirteen differentially expressed miRs could separate LGMD vs control group by hierarchical clustering. Most of differentially expressed miRs in LGMD patients were up-regulated (miR-122-5p, miR-122b-3p, miR-6511a-3p, miR-192-5p, miR-574-3p, mir-885-3p, miR-29a-3p, miR-4646-3p, miR-203a-3p and miR-203b-5p) whilst only three of sequenced miRs were significantly down-regulated (miR-19b-3p, miR-7706, miR-323b-3p) when compared to matched controls. Bioinformatic analysis of target genes revealed cell cycle, muscle tissue development, regeneration and senescence as the most affected pathways. Four of these circulating miRs (miR-122-5p, miR-192-5p, miR-19b-3p and miR-323b-3p), together with the myomiR miR-206, were further analysed by qPCR in LGMD, DMD and FSHD. The receiver operating characteristic curves (ROC) revealed high area under the curve (AUC) values for selected miRs in all groups, indicating that these miRs have good sensitivity and specificity to distinguish LGMD, DMD and FSHD patients from healthy controls. miR-122-5p, miR-192-5p and miR-323-3p were differentially expressed compared to matched-controls in all groups but apparently, each type of muscular dystrophy showed a specific pattern of miR expression. Finally, a strong correlation between miRs and biochemical data was only found in LGMD patients: while miR-192-5p and miR-122-5p negatively correlated with CK, miR-192-5p positively correlated with vitamin D3 and ALP.

**Conclusions:**

Although limited by the small number of patients included in this study, we propose here a specific combination of circulating miR-122-5p/miR-192-5p/miR-323-3 and biochemical parameters as a potential molecular signature whose clinical value for LGMD patient prognosis and stratification should be further confirmed in a larger cohort of patients.

**Supplementary Information:**

The online version contains supplementary material available at 10.1186/s13023-022-02603-3.

## Background

Limb-girdle muscular dystrophies (LGMD) are a heterogeneous group of rare genetic disorders that primarily affect skeletal muscle and are characterized by progressive weakness and atrophy of the pelvic and shoulder girdle muscles. More than 30 different genetic subtypes of LGMD have been identified and initially classified into autosomal dominant (LGMD1) or recessive (LGMD2) disorders. Up to eight autosomal dominant (A-H) and 23 autosomal recessive (A-W) LGMD variants were mapped [1]. However, in 2017, the classification was revised naming the autosomal dominant LGMDs as D and numbering them from 1 to 5, and the recessive forms as R with numbers from 1 to 23 [2, 3]. The updated definition of LGMDs included dystrophies with proximal or disto-proximal presentation, showing fiber degeneration on muscle histology, elevated serum creatine kinase (CK) and degenerative changes on muscle MRI images including fibro-fatty infiltration [3].

LGMD patients can show a wide array of phenotypical manifestations with a variable clinical course of the disease. In fact, LGMDs are characterized by a broad spectrum of muscles involved and clinical manifestations ranging from severe phenotype with early-onset and rapid progression, usually occurring in childhood-onset disease, to a milder form which is usually associated to late-onset disease [4]. In this sense, elucidating the molecular mechanisms underlying LGMD subtypes can further help to understand phenotypic variability among patients.

To date, most proteins and mechanisms underlying the development of LGMD have been identified, including structural defects in the sarcolemma, deficiencies in sarcolemma repair and defects in sarcomere remodelling, as well as impaired cytoskeletal structures and cytoskeleton-membrane interactions. A deeper understanding of the disease pathways points out to defects in mechanical signal transduction and mitochondrial function. The former frequently includes the alteration in the glycosylation pathway which encompasses the dystroglycanopathies. These subtypes of LGMD are caused by impaired glycosylation of dystroglycans and include genetic mutations in *FKRP, POMT1, FKTN, POMT2, POMGNT1, ISPD, POMGNT2*, and *GMPPB*. Alterations in mitochondrial function have also been addressed as key contributors to explain the pathophysiology of LGMDs. This may occur through altered energy production, impaired Ca^2+^ homeostasis or activation of apoptosis, although the exact patho-mechanism for each LGMD subtype remains to be elucidated [5].

MicroRNAs (miRs) are small 21–24 nucleotide RNAs that regulate gene expression by binding to their target mRNAs, affecting multiple signaling pathways across multiple tissues. miRs are dynamically regulated and change its expression both, in physiological and pathological processes. miRs are frequently altered in conditions such as cancer, cardiovascular, metabolic diseases, and neuromuscular disorders [6]. miRs, known to be secreted by various cell types, are remarkably stable in circulating body fluids due to their vesicular protection from ribonucleases.

miRs are actively involved at various stages of embryonic and adult myogenesis, by modulation of stem cells during adult life. In fact, myomiRs are a group of miRs with a role in orchestrating skeletal myogenesis, being miR-1 and miR-133 the main members of this family [7]. Nevertheless, more than one hundred miRs are known to modulate their expression in murine myoblasts and in myotubes in vitro, which suggests that those miRs are critical regulators of skeletal muscle differentiation [8]. Moreover, miRs are part of the epigenetic regulation of gene expression; it is well-established that the interplay between miRs and other epigenetic factors such as DNA methylation, histone acetylation/deacetylation or histone methylation plays a role in myogenesis, and myoblast differentiation during muscle development [9].

Furthermore, numerous pathologies are associated with changes in the amount of miRs either in tissue samples or in levels of serum circulating miRs. The use of miRs as a diagnostic tool for muscular disorders was suggested in 2007 by Eisenberg et al. who demonstrated a differential miR profile in muscle biopsies from ten different primary muscular disorders [10]. Recently, miRs have been isolated from whole serum and muscle biopsies to identify unique diagnostic signatures for specific neuromuscular disease states. In this sense, Matsuzaka et al. evaluated in serum exosomes of a small cohort of LGMD patients the levels of those miRs previously identified in Duchenne muscular dystrophy (DMD) [11]. Following those previous studies, other miRs such as miR-1, miR-133a, miR-206, miR-21, miR-31, miR-142-3p, miR-378a-3p, miR-149-5p or miR-193b-3p have been identified in several neuromuscular diseases and recognized as “dystromiRs” [12]. More recently, Pegoraro and Angelini [13], showed that circulating miR-206 levels were significantly elevated in LGMD patients compared to healthy controls. However, although these studies further increase our knowledge of the LGMD pathophysiology, the rare condition of this disease limits the size of samples used to stablish a molecular signature. The identification of LGMD-specific miRs, rather than a redundant task would further contribute to the design of a more accurate miR signature for LGMD patient stratification, prognosis, or treatment-response.

In the present study we have first used whole miRNome sequencing in plasma samples from six LGMD patients and their matched-controls to identify a specific signature for the disease. We further investigated whether this miR signature could be a potential biomarker for the differential diagnosis among muscular dystrophies. Accordingly, the levels of deregulated miRs were validated in LGMD and tested in two other common neuromuscular disorders such as DMD and facioscapulohumeral muscular dystrophy (FSHD).

## Results

### LGMD patients cohort

Six Caucasian LGMD patients (three males and three females) from different families were enrolled in the miRNome study. All of them were symptomatic at the time of data collection. Regarding the genetic diagnosis, three out of six LGMD patients had mutations in CAPN3 gene, two of them were in TTN and one patient showed SGCA mutation. Table 1 shows the clinical and biochemical characteristics, and most common symptoms of these patients. In summary, proximal limb weakness was the most frequent symptom observed in five out of the six patients. Only two patients were wheelchair-bound and only one showed cardiomyopathy, while none of them had the respiratory function compromised. Serum CK levels were elevated 4–50 times the normal values in five out of six patients, widely ranging between 877 and 9827 IU/L (normal values < 195 IU/L).

**Table 1:** Clinical and biochemical features of LGMD patients

Patient #	LGMD 1.1	LGMD 6.1	LGMD 7.1	LGMD 8.1	LGMD10.1	LGMD 16.1
Muscular distrophy	LGMD2J/LGMDR10	LGMD1I/LGMDD4	LGMD2D/LGMDR3	LGMD1I/LGMDD4	LGMD2J/LGMDR10	LGMD1I/LGMDD4
Mutated gene	NM_001267550.2(TTN)	NM_000070.3(CAPN3)	NM_000023.4(SGCA)	NM_000070.3(CAPN3)	NM_001267550.2(TTN)	NM_000070.3(CAPN3)
Sex	F	M	M	F	M	F
Early/late onset, age	Late, 28	Early, 18	Early, 9	Early, 9	Early, 7	Early,7
Age last evaluation	51	40	24	18	11	20
Limb weakness^a^	3	3	1	1	1	2
WCB^b^, age	No	Yes, 18	Yes, 23	No	No	No
Functional System Score (FSS)^c^	1	3	2	1	2	2
Cardiopathy	No	No	Yes	No	No	No
Non-invasive ventilation required	No	No	No	No	No	No
Muscle biopsy	Yes	Yes	Yes	No	No	No
Calcium (mg/dL)	10	9.2	9.5	9.7	9.5	9.8
iP (mg/dL)	4.2	4.2	3.4	3.9	43	3.8
Vit D3 (ng/mL)	20.5	10.2	17.9	0.4	47.4	17.1
PINP (ng/mL)	–	33.2	40.2	36.3	460	70.2
ALP (IU/L)	–	55	68	56	321	56
PTH (pg/mL)	65.4	48	41.9	30	25	27.9
PG (ng/mL)	0.15	0.26	0.47	0.4	0.21	0.84
FSH (mU/mL)	133	2	2.4	0.1	2.1	4.7
LH (mU/mL)	102	3	2.8	0.1	3.2	18.8
CK (IU/L)	877	2798	1922	9827	124	5733

^a^1: Proximal; 2: distal; 3: both proximal and distal

^b^*WCB* wheel-chair bound

^c^1: Fast/Severe; 2: moderate/progressive; 3: mild/long-term

### Identification of differentially expressed miRNAs by small RNA-sequencing

**Fig. 1:**
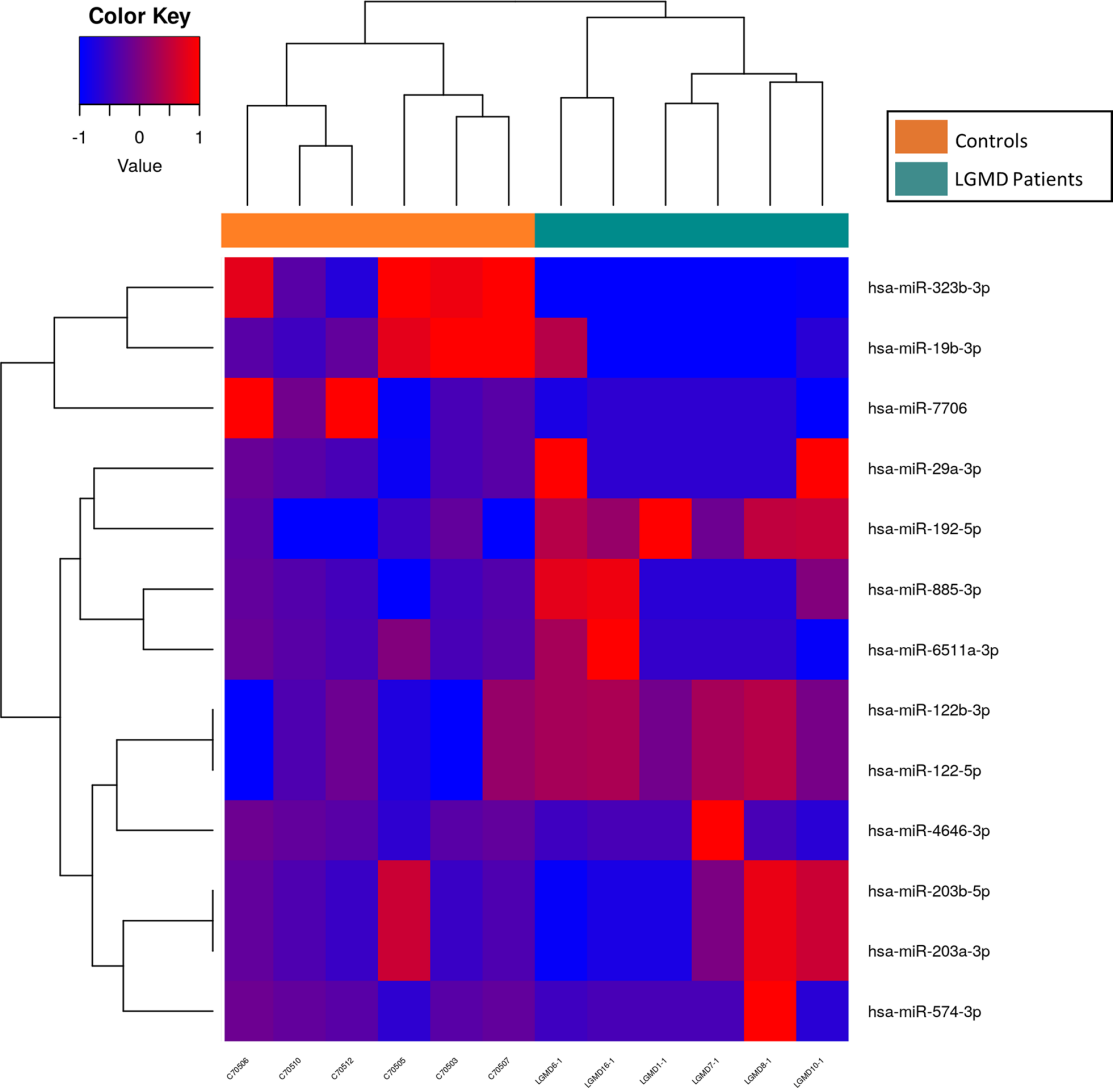
Heatmap of miRNAs in LGMD patients and healthy controls. Hierarchical clustering of samples by the logCPM (counts per million) for those miRs considered significant (FDR < 0.05) in LGMD Patients (*n* = 6) vs Controls (*n* = 6) using the test edgeR. The logCPM values were transformed and centered by mean value from − 1 to 1 for better visualization. UP = overexpressed in LGMD patients, value closer to 1 (red). DOWN = under-expressed in LGMD patients, value closer to − 1 (blue)

The analysis of the whole miRNome in plasma showed a distinct clusterization of LGMD patients (*n* = 6) and control samples (*n* = 6). It showed differential expression of 13 miRs between the two groups (FDR < 0.05). While seven miRs (miR-122-5p, miR-122b-3p, miR-6511a-3p, miR-192-5p, miR-574-3p, mir-885-3p, miR-29a-3p, miR-4646-3p, miR-203a-3p, miR-203b-5p) were upregulated to some extent, only three of sequenced miRs were significantly down-regulated (miR-19b-3p, miR-7706, miR-323b-3p) in LGMD patients when compared to matched-healthy controls. Finally, LGMD and control samples were efficiently grouped by clustering heatmap of up- and down-regulated miRs (Fig. 1).

### Functional enrichment analysis of miRNA targets in LGMD patients

**Fig. 2:**
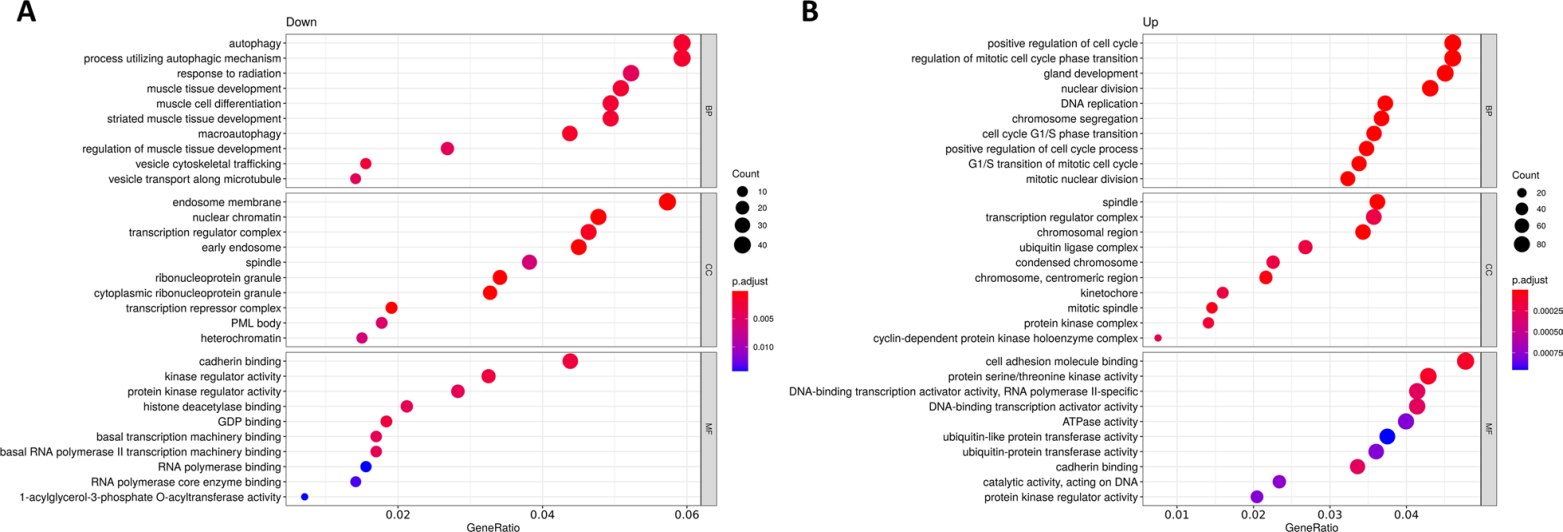
Gene Ontology (GO) over-representation analysis (ORA) dotplot of circulating miRNAs from LGMD patients. Functional analyses of the significantly **A** down-regulated and **B** up-regulated circulating miRs found in LGMD patients versus control. GO dotplot: the y-axis represents the term where genes are enriched, and the x-axis represents the ratio of term genes to the total genes in the pathway. The colour of the dot represents the *P*-value (Fisher’s exact test performed in ORA), and the size of the dot represents the number of gene enrichment (counts per million miRs of each path)

**Fig. 3:**
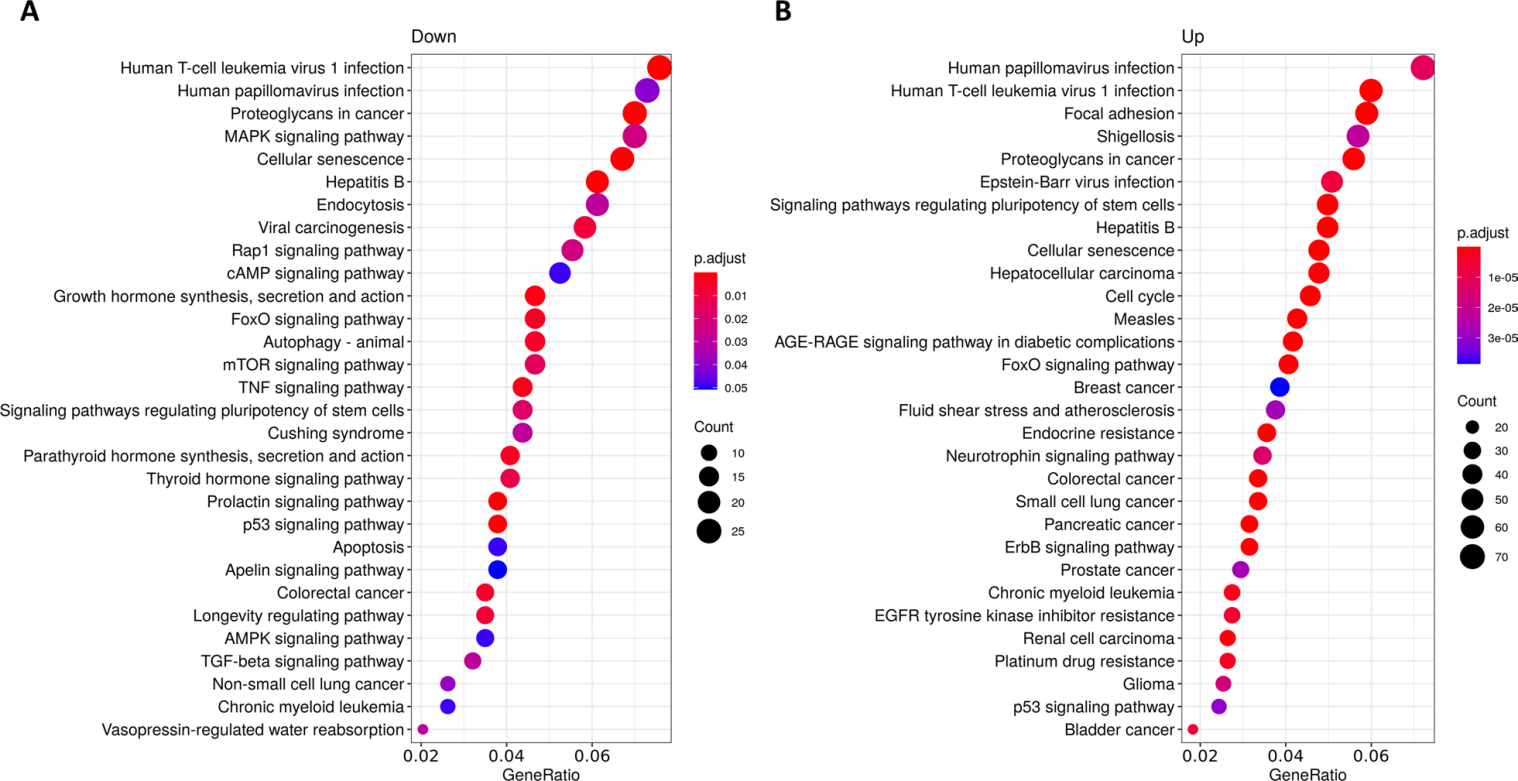
KEGG pathway enrichment analysis of circulating miRNAS from LGMD patients. Pathways enrichment of target genes of **A** down-regulated and **B** up-regulated miRs. KEGG pathways are classified according to the gene ratio: number of genes participating in the current KEGG pathway to number of genes annotated as participants of any KEGG pathway. The colour of the dot represents the *P*-value (Fisher’s exact test performed in ORA), and the size of the dot represents the number of gene enrichment (counts per million miRs of each path)

To clarify the role of the identified miRs in LGMD, we analyzed molecular networks regulated by differentially expressed miRs. Over-representation analysis (ORA) using GO terms and KEGG database was used to study the functional enrichment of target genes of up- and down-regulated miRs. A total of 939 GO terms (*p* < 0.05) were obtained when compared LGMD patients to matched-healthy controls. As expected, most of them associated to up-regulated miRs (916 for up-regulated and 23 for down-regulated miRs). Regarding KEGG pathways, a total of 156 terms were found (114 for up-regulated and 24 for down-regulated miRs). The most significative GO terms and KEGG pathways are showed in Figs. 2 and 3, respectively.

Notably, the biological processes identified in the GO analysis for down-regulated miRs (Fig. 2A) were involved in muscle tissue development and regeneration (autophagy, muscle cell differentiation). On the other hand, cell-cycle related pathways (DNA replication, chromosome segregation, nuclear division, or cell cycle phase transition) were the most representative pathways for targets of up-regulated miRs (Fig. 2B). Importantly, among the KEGG pathways in the group of down-regulated miRs we found cellular senescence, autophagy, and an important enrichment in signal transduction pathways such as, MAPK, cAMP, FoxO, mTOR, TGF-beta and signaling pathways regulating pluripotency of stem cells (Fig. 3A). KEGG pathways obtained with up-regulated miRs included focal adhesion, proteoglycans in cancer, signaling pathways regulating pluripotency of stem cells, cellular senescence, cell cycle, FoxO signaling pathway or ErbB signaling pathway (Fig. 3B). Remarkably, target genes of up-regulated miRs in LGMD patients were significantly enriched in cancer-related pathways (hepatocellular carcinoma, breast cancer, colorectal cancer, small cell lung cancer, pancreatic, prostate or bladder cancer).

### Validation of differentially expressed miRNAs in LGMD patients

**Fig. 4:**
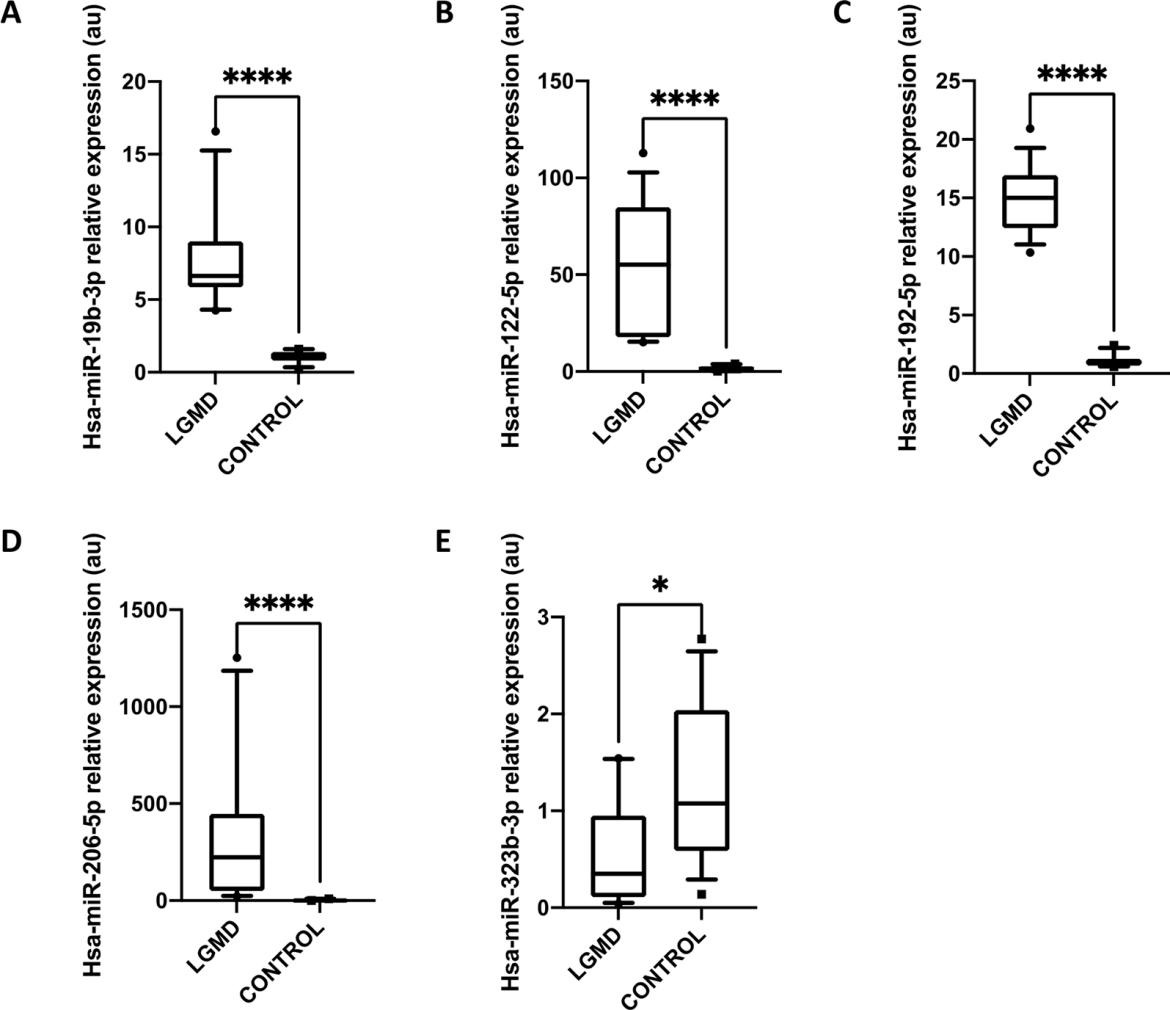
Differential expression of selected miRNAs in plasma of LGMD patients. Boxplot showing fold expression levels of **A** miR-19b-3p; **B** miR-122-5p; **C** miR-192-5p; **D** miR-206-5p; and **E** miR-323b-3p in LGMD patients (*n* = 6) and healthy controls (*n* = 6). Expression of miR-191-5p was used for normalization. Statistically significant differences were determined using Mann–Whitney U tests. All *P*-values were two-tailed: ****p < 0.0001, ***p < 0.001, **p < 0.01 or *p < 0.05 vs matched controls

Four of the differentially expressed miRs detected by NGS were validated by RT-qPCR including a representative selection of up- and down-regulated miRs. Additionally, dystromiR miR-206 with a key function in skeletal muscle was included as a positive control. Relative expression levels for each miR were calculated, using miR-191-5p as endogenous reference due to its stable counts and threshold cycle (Ct) values in all samples analyzed by NGS and RT-qPCR, respectively. Analysis of these miRs included six LGMD patients and six age- and sex-matched controls. Three miRs were significantly up-regulated in LGMD patients: miR-19b-3p, miR-122-5p, and miR-192-3p compared to healthy controls (Fig. 4). The dystromiR miR-206 already described to be up-regulated in other studies and other neuromuscular disorders, was also statistically up-regulated in these patients. On the other hand, the expression of miR-323-3p was statistically downregulated in LGMD when compared to control subjects, confirming the results obtained in the miRNome analysis (Fig. 4E).

### Specificity of miRNA-signature in LGMD patients

To elucidate whether these differentially expressed miRs were specific to LGMD neuromuscular disorder or might also be involved in the pathology of other muscular dystrophies, we extended the study to patients affected by two of the most common muscular dystrophies, such as DMD (*n* = 5) and FSHD (*n* = 4). Again, new specific age- and sex-matched controls were used for the experiments with these two new groups of patients. The clinical and pathological characteristics of these patients are detailed in Additional file 1: Table S1 and Additional file 2: Table S2 for DMD and FSHD cohorts, respectively. Overall, DMD patients showed higher levels of serum CK, worse disease progression (four out of five had wheelchair dependency) and more than half of them showed cardiac dysfunction.

**Fig. 5:**
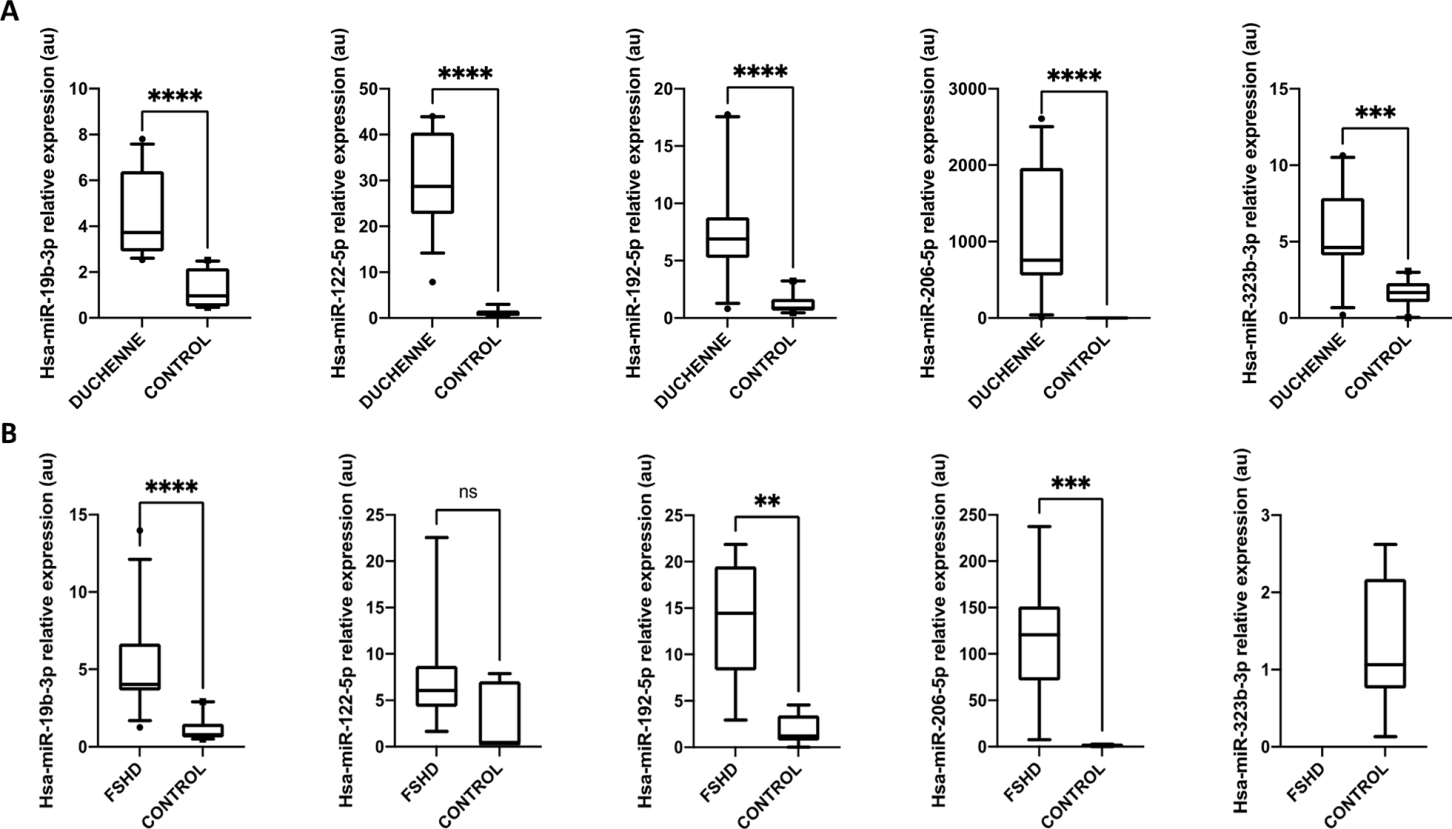
Differential expression of selected miRNAs in plasma of DMD and FSHD patients. Boxplot showing fold expression levels of indicated miRs in **A** DMD patients (*n* = 5) and matched controls (*n* = 5) and in **B** FSHD patients (n = 4) and healthy controls (*n* = 4). Expression of miR-191-5p was used for normalization. Statistically significant differences were determined using Mann–Whitney U tests. All *P*-values were two-tailed: ****p < 0.0001, ***p < 0.001 and **p < 0.01 versus matched controls

The five miRs validated in LGMD were also studied by qPCR in these two groups. In DMD patients miR-19b-3p, miR-122-5p, miR-192-3p and miR-206 were overexpressed as it happened in LGMD patients (Fig. 5A). However, miR-323-3p was up-regulated in DMD patients while in LGMD was down-regulated. This miR has been related to cardiomyopathy and cardiac fibrosis [14, 15], thus it is not surprising to find it dysregulated in DMD, since those patients usually show heart disease associated to the neuromuscular disorder [16].

In the FSHD cohort miR-19b-3p, miR-192-3p, and miR-206 were also up-regulated, as it happened in both LGMD and DMD patients (Fig. 5B). However, miR-122-5p did not show any statistical change compared to matched controls. Surprisingly, miR-323-3p which was differentially expressed in LGMD vs DMD patients, showed a dramatic variation in FSHD patients, where the miR was undetectable compared to their matched controls (Fig. 5B).

Overall, these data seem to indicate that miR-323-3p is differentially expressed in the three neuromuscular dystrophies; being down-regulated in LGMD, up-regulated in DMD and undetected in FSHD when compared to their respective matched-controls. However, although miR-122-5p and miR-192-5p were up-regulated in the three groups, the magnitude of their expression highly differed among the diseases (Additional file 3: Table S3). In brief, miR-122-5p and miR-206-5p increased to a lesser degree in FSHD, whereas miR-192-5p was expressed to lower levels in DMD patients.

**Fig. 6:**
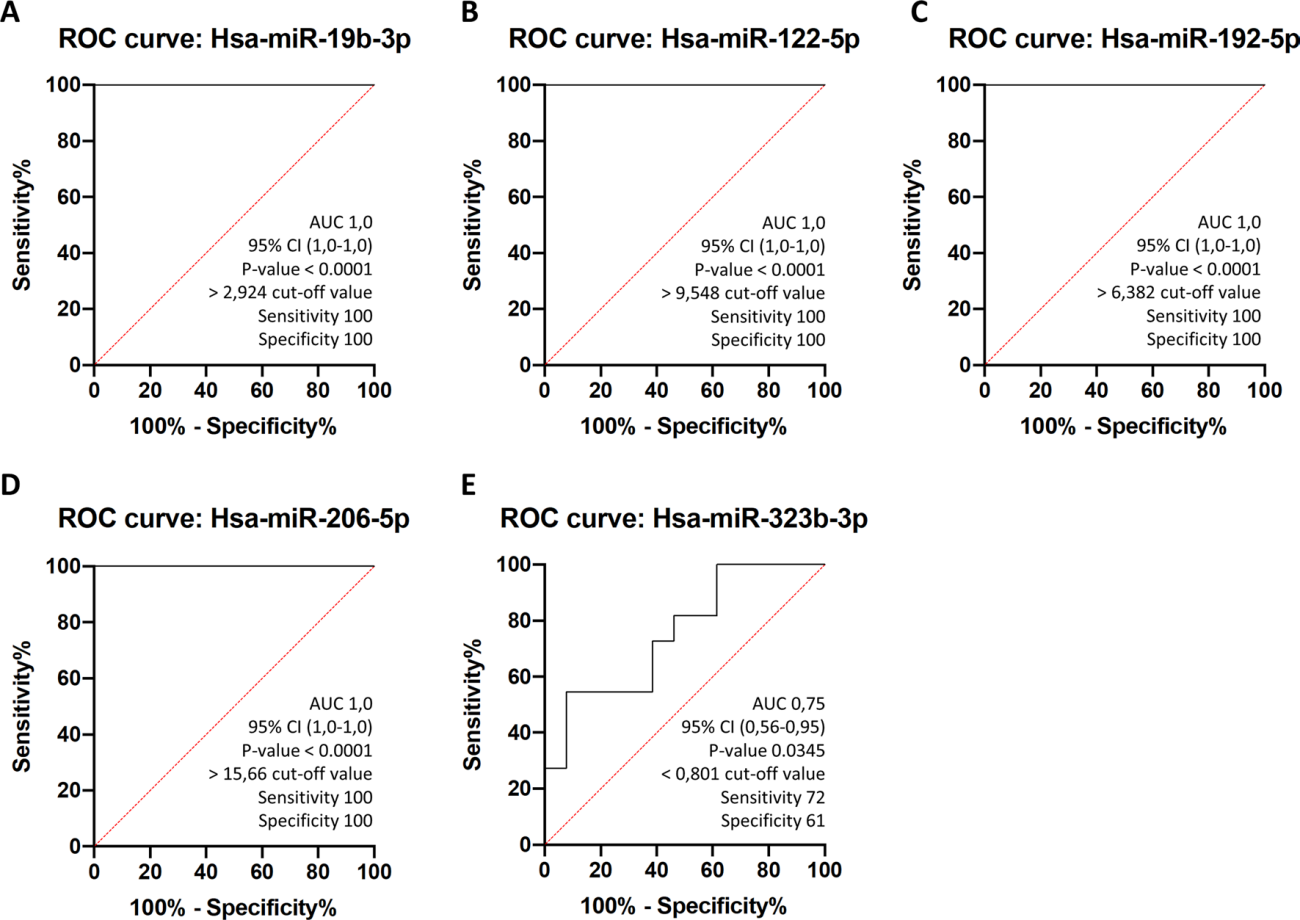
Receiver operating curves (ROC) of circulating miRNAs separate LGMD patients from healthy controls. ROC curves were represented for **A** miR-19b-3p **B** miR-122-5p, **C** miR-192-5p, **D** miR-206-5p and **E** miR-323b-3p using the whole cohort of healthy participants (controls) (*n* = 6) and LGMD patients (*n* = 6). AUC, % CI, cut-off and *P*-values for indicated miRs are included in their respective ROC curves

To evaluate the diagnostic value of circulating miRs, the receiver operating characteristic curves (ROC) for the four miRs were obtained in all the groups. The results revealed that all of them could be used to distinguish LGMD patients from healthy subjects. The area under the ROC curve (AUC), standard error, 95% confidence interval (CI), fold change cut-off value, sensitivity, and specificity for each miR are shown in Fig. 6. Among miRs analyzed to elucidate their potential as biomarkers for LGMD diagnosis, except for miR-323b-3p, all of them showed excellent ROC curve parameters (AUC = 1, sensitivity = 100%, specificity = 100%), indicating that these circulating miRs have good sensitivity and specificity in distinguishing LGMD patients from healthy controls, and may be used as new serum biomarkers for LGMD management.

Regarding ROC curves in DMD and FSHD patients (Additional file 5: Fig S1), high specificity and sensitivity were also obtained for most of the miRs analyzed (AUC > 0.9 for all DMD and AUC > 0.93 for most FSHD).

### Correlation between biochemical parameters and circulating miRNAs expression in LGMD patients

**Fig. 7:**
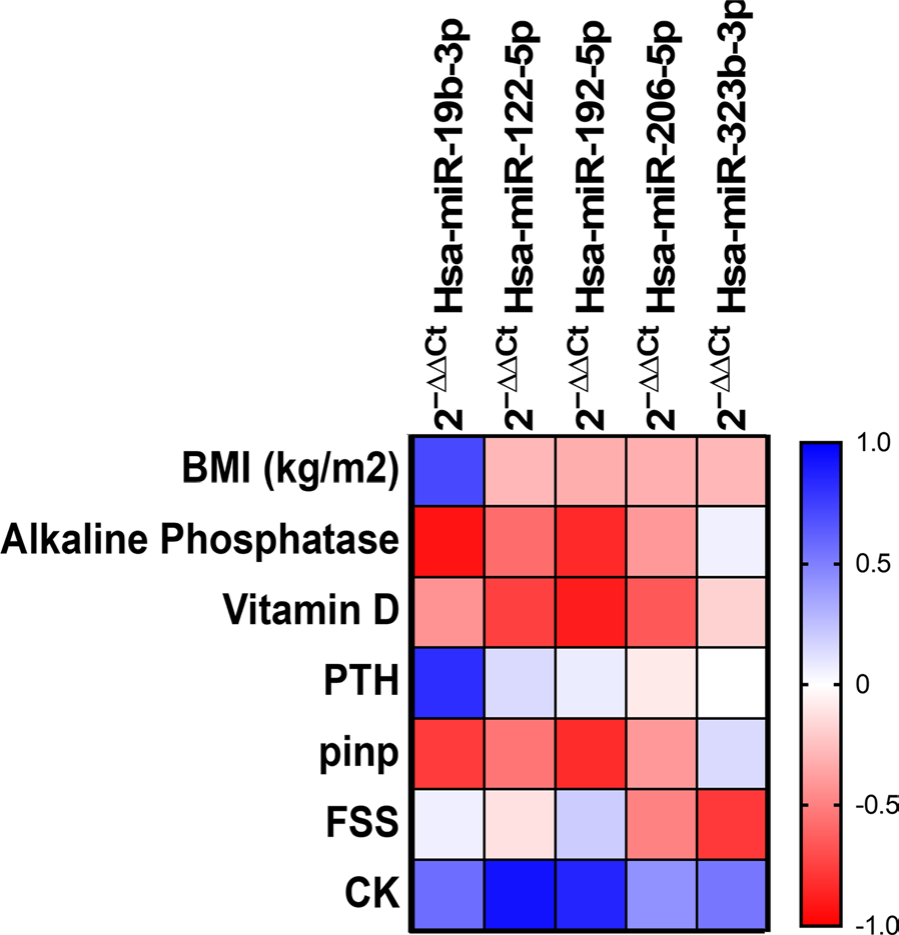
Correlation between clinical and biochemical parameters and circulating miR in LGMD patients. Spearman's rank correlations coefficients (− 1 to + 1) among the biochemical variables and circulating miRs in LGMD patients. Red color indicates a negative correlation and blue color indicates a positive correlation between compared parameters

To identify a more specific miR signature for LGMD we explored the correlation of miR expression and clinical or biochemical parameters measured in serum samples from LGMD patients. We observed that miR-192 negatively correlated with CK, but positively correlated with vitamin D3 and ALP levels. miR-19 levels also showed a positive correlation with ALP, but negative with PTH levels. Finally, a strong negative Spearman’s correlation between miR-122-5p and CK levels was obtained (Fig. 7; Additional file 1: Table S4). Importantly, no correlation between any miR and biochemical parameters was found in patients affected by DMD and FSHD (results not shown).

In addition, we also performed a comparison study to evaluate the differences between clinical status (wheelchair use, difficulties to walk, Functional System Score (FSS), etc.) and the relative expression of miRs, but no significant differences were found.

## Discussion

Muscular dystrophies are a group of rare and heterogeneous neuromuscular disorders sharing few common characteristics, frequently leading to patient misdiagnosis. Different genes are responsible for the various types of muscular dystrophies and the development of the disease. Currently, these patients are diagnosed based in clinical phenotype, histopathological analysis, or exome sequencing/genetic studies [17, 18]. miRs have been shown to be involved in the cellular response to dysregulated proteins in most diseases. The identification of miRs specifically associated to muscle disorders could improve the diagnosis, prognosis, or follow-up of drug-pharmacodynamics in these patients.

In this sense, an early study in muscle-tissue samples tried to identify muscle-specific miRs hypothetically underlying LGMD, FSHD or DMD diseases [10]. Although, most of predicted miRs were dysregulated in various dystrophies or even in non-dystrophic muscle diseases, some of them were found to be expressed in a disease-specific manner. MyomiRs dysregulation was first observed in muscle biopsy but also in plasma samples of patients with muscular dystrophies [12]. Consequently, myomiRs have been proposed as biomarker candidates for muscular dystrophies and as potential tools to monitor the evolution of muscle diseases [19]. Unfortunately, most of these studies demonstrate just an increase of several miRs levels in serum from patients with muscular dystrophies compared to healthy subjects, without any correlation to the functional status. In fact, only few publications showed a correlation between serum levels of these myomiRs (such as miR-1, miR-133 and miR-206) and motor functions, muscle strength or myocardial affection in DMD [20, 21]. Another paper has also proposed miR-206 as a candidate serum biomarker for FSHD [22]. Furthermore, miR-206 levels have been correlated to the progression of muscle wasting in myotonic dystrophy type 1. It seems that miR-206 is a biomarker of muscle wasting shared by different muscular dystrophies [23].

However, although previous studies have undoubtedly broadened our understanding of these rare neuromuscular diseases, there are not so many evidences of miRs as biomarkers for other rare forms of muscular dystrophies, such as LGMD and, the current clinical needs of these patients remain uncovered. The progression in our knowledge of LGMD has revealed the need for a non-invasive method for the identification of new biomarkers for patient stratification as well as for the development of clinical test to monitor disease progression and drug pharmacodynamics. It is important to highlight that in all LGMD subtypes the main source of circulating miRs is most likely proximal limb muscle [12]. However, other phenotypes such as calf hypertrophy, facial weakness, macroglossia, myoglobinuria, changes in muscle fat fraction, cardiomyopathies, white matter brain lesions, epidermolysis bullosa or congenital pyloric atresia are also observed during disease progression in different LGMD subtypes [24]. Accordingly, different tissues in the organism could release miRs to the bloodstream of LGMD patients. The analysis of circulating proteins and miRs could be crucial when trying to identify potential biomarkers for either LGMD diagnosis or disease progression. Here we tried to identify a molecular signature of circulating miRs with a potential use for LGMD patient stratification and management.

A total of 13 miRs were found to be dysregulated in LGMD patients when compared to age-matched control group. Recently reported data showed a more severe progression for LGMD2B males than females [25]. However, in our study no significant sex-related differences were found among identified miRs in LGMDD and LGMDR patients, suggesting a sex hormone-independent pathway for miR dysregulation. Alternatively, since a limitation for the current study is the low number of LGMD samples, a sex-related dysregulation of miRs might be observed when using a larger cohort of LGMD patients to compare disease progression according to genotype [25].

Our KEGG analysis revealed a functional enrichment of signal transduction pathways such as MAPK, TGF-β, cAMP, TNF, FoxO, mTOR or AMPK mostly in gene-targets of down-regulated miRs. Most of them, were previously described to be involved in skeletal muscle atrophy and weakness [26, 27]. However, FoxO signal transduction pathways or other pathways such as, human T-cell leukemia virus 1 infection and human papillomavirus infection were targeted by both, down- and up-regulated miRs, suggesting a fine-tuned regulation of these pathways in LGMD. Interestingly, up-regulated miRs were strikingly enriched in target genes related to a variety of types of cancer including colon, breast, lung, bladder, kidney, liver, pancreas, prostate or glioma. So far, to our knowledge increased cancer risk in LGMD patients has not been described. However, a growing number of evidences links other neuromuscular dystrophies such as DMD to tumorigenesis (including sarcomas, carcinomas, melanomas, lymphomas, leukaemia, or brain tumors) [28]. An in silico meta-analysis unveiled a common transcriptome signature between FSHD and malignant tumors and demonstrated that FSHD and DMD share the same cancer-related genes [29]. Aneuploidy, double stranded DNA breaks (DSBs) and increase incidence of tumors in mice models of muscular dystrophies, including LGMD has been reported [30]. Moreover, aneuploidy and DSBs, both considered as pre-neoplastic events, were also observed in human samples of LGMD. Finally, unrepair DSBs can promote cellular senescence [31], another pathway significantly enriched in target-genes of up-regulated miRs, which could diminish the regenerative capacity of muscle cells. All in all, although the incidence of cancer in LGMD seems to be accidental, data in the literature and herein reinforce the need for further investigation to unveil a putative role of dysregulated miRs on increased risk of cancer during LGMD progression.

Except for miR-19b and miR-192, previously described to be up-regulated in muscle-tissue from FSHD, LGMD and LMNA-mutated patients with muscular dystrophy [10, 32], the rest of circulating miRs identified by smallRNA-seq in the current study were never described in muscular dystrophies or as muscle-specific miRs. In agreement with this, a high percentage (40%) of miRs differentially expressed in muscle-tissue from several neuromuscular dystrophies including LGMD, had not been previously described as muscle-specific miRs [10]. In addition, circulating miRs might also have a non-muscular origin and reflect the secondary response to muscle damage [24]. Finally, different techniques or biological samples might detect different miRs. For instance, the well-known myomiR-206 (also proposed as dystromiR [11–13]) was not identified by smallRNA-seq in the present study but could be detected by qPCR among up-regulated circulating miRs in LGMD, FSHD and DMD patients. Adding new miRs to those already identified could facilitate the design of a more accurate panel with diagnostic/prognostic value for LGMD patients.

Herein four miRs were found to be differentially expressed in LGMD patients versus matched controls, but most importantly, miR-122, miR-192 and miR-323 levels seem to discriminate LGMD from DMD or FSHD dystrophies. The analysis of single miR expression seems to be an inefficient and unspecific approach for LGMD diagnosis. To find a specific combination of biomarkers able to dissect LGMD from FSHD and DMD has been a major challenge. Factors common to several muscular dystrophies could be the consequence of muscle degradation rather than the underlying cause or specific outcome of the disease. In this sense, it has been shown that serum proteins cannot discriminate among neuromuscular diseases [33].

A strong correlation between some circulating miRs and biochemical variables such as CK, was found in LGMD patients. In this sense, we would like to highlight the negative correlation between miR-122 and miR-192 with CK levels. It is long ago known that CK levels can increase during disease progression and severity [2, 33]. Unfortunately, high CK levels are not equally observed in all muscular dystrophies or even in the same type of muscle dystrophy [34, 35]. Consequently, its usefulness as a specific biomarker of LGMD or any other neuromuscular disease could not be clinically considered. It could be argued that CK levels are known to increase with age or physical exercise [35, 36] and consequently, this variable could lead to data misinterpretation. However, age-matched controls were used in this study and increased exercise seems not to be a cause of high CK levels in patients with reduced mobility versus healthy controls. On the other hand, LGMD patients may often develop scoliosis and thus, clinical parameters related to bone tissue were also analyzed. Several studies have shown the importance of miRs for osteoblast differentiation and function, as major regulators of bone metabolism [37]. Besides, it has already been described that myomiRs regulate important genes in key pathways for bone tissue homeostasis. In this sense, miR-206 has already been reported to regulate osteoblastogenesis [38], miR-19b-3p, stimulates osteogenic differentiation [39] and recent clinical studies have revealed that miR-122-5p is associated with bone deterioration being up-regulated in osteoporotic patients [40]. Regarding other bone parameters, a significant correlation was found for both vitamin D and ALP and myomiRs miR-133 and miR-206 in elderly women following resistance-type training, reflecting that training improves osteoporotic markers [41]. Interestingly, miR-192-5p has been shown to regulate genes involved in vitamin D metabolism [42]. In agreement with these previous results, we found a strong correlation for some circulating miRs and vitamin D3, ALP or PTH in LGMD patients. All in all, this suggest that myomiRs might be involved in bone health status. Nonetheless, the combination of circulating miRs, CK and vitamin D3 levels to test the progression of a specific disease has never been used before. In the future, the predictive value of combined miRs and biochemical parameters might be assessed in a prospective study, in which this molecular signature could be related to disease-progression.

## Conclusion

We propose here a specific combination of easy-to-obtain molecular and biochemical parameters with potential value for diagnosis/prognosis of LGMD patients versus the most frequent neuromuscular dystrophies DMD and FSHD. The rare condition of these diseases limits the obtention of patients’ samples. Consequently, a limitation for the current study is the low number of samples analysed. Although aware of this limitation, we strongly believe that data presented here deserves further investigation in a larger cohort of patients which should finally confirm or discard the clinical value of this molecular signature. All in all, these findings can undoubtedly increase our knowledge of miRs as epigenetic modulators of muscular dystrophies, but most importantly, provide a putative combination of miRs and biochemical parameters as non-invasive biomarkers for these muscular disorders.

## Materials and methods

### Study design and population

A total of six patients diagnosed of LGMD at *Hospital Universitari i Politècnic La Fe* de Valencia (Spain) were used for the miRNome analysis. Two cohorts of patients of DMD (*n* = 5) and FSHD (*n* = 4) were also included in the study for further validation of results. Informed written consent was obtained from all participants. Blood samples were taken by venipuncture in the *Hospital Universitari i Politècnic La Fe* and immediately transported, processed, and stored according to the standard operating procedures in the CIBERER Biobank for further research (www.ciberer-biobank.es). Age and gender-matched healthy controls (*n* = 15) for all groups were provided by IBSP-CV Biobank and processed in the same way.

The inclusion criteria for the study were: (1) genetically confirmed diagnosis of LGMD, DMD or FSHD by the identification of gene mutations and (2) availability of clinical records and follow-up. Exclusion criteria were: smoker, infectious process during the extraction, other neurological or neoplastic pathologies, and for control group any congenital syndrome pathology.

### Clinical course and examination

All subjects underwent a detailed clinical examination during the screening visit to the Traumatology Department in the *Hospital Universitari i Politècnic La Fe*. Clinical data included gender, age, disease onset and progression, limb weakness (either proximal or distal muscle weakness), wheelchair dependency or respiratory/cardiac related symptoms. Besides, a survey recording the Functional System Score [43] (FSS) was also obtained from all patients. Muscle biopsies data were used either as a diagnostic standard or as functional evidence of pathogenicity, when available.

The biochemical parameters analysed in blood samples were: Creatinine Kinase (CK) levels, alkaline phosphatase (ALP), Procollagen 1 Intact N-Terminal Propeptide (PINP) and hormonal status including, progesterone (PG), Follicle-stimulating hormone (FSH), Luteinizing Hormone (LH), Parathyroid Hormone (PTH) and vitamin D3.

### RNA extraction and quantification

Blood samples were collected from LGMD, DMD and FSHD patients and healthy participants in EDTA tubes. Each sample was centrifuged to separate plasma; then, a second centrifugation was performed at 16,000*g* and 4 °C for 10 min. Finally, plasma samples were stored at − 80 °C until RNA extraction. Cell-free total RNA (including miRs) was isolated from 400 µL of plasma using the miRNeasy Serum/Plasma kit (Qiagen, Hilden, Germany), following the manufacturer’s protocol. RNA was eluted with 25 µL of RNase-free water. The concentration of total cell-free RNA (including miRs) was quantified using NanoDrop™ One Microvolume UV–Vis Spectrophotometer (Thermo Scientific Inc, Waltham, MA, USA).

### Library preparation and small RNA-sequencing

Small RNA libraries were generated and indexed using the Lexogen’s Small RNA-Seq Library Prep Kit for Illumina sequencing (Lexogen GmbH Campus Vienna Biocenter 5, 1030 Vienna, Austria). Briefly, for library generation 3 ‘-adapter was ligated to RNA and their excess removed by column purification. Next, 5’-adapter was ligated and the RNA, flanked by 5 ‘and 3’ adapters, was converted into cDNA. Library amplification was performed to add the complete adapter sequences necessary for the generation of clusters and to assure enough material for quality control and sequencing. External i7 indexes were added during this step to enable multiplexing of libraries for sequencing. The library product was then purified to remove interfering PCR components. Single-end sequencing was performed on Illumina NextSeq550 platform (Illumina, San Diego, CA, USA) on High Output 1 × 50pb RUN (NextSeq 500/550 High Output v2 75 cycles kit, FC-404-2005).

### Differential expression analysis

The quality of the Illumina raw sequences, pre-processing, quality control and normalization was assessed using FastQC software (https://www.bioinformatics.babraham.ac.uk/projects/fastqc/). Sequence reads were trimmed to remove sequencing adapters and low-quality bases using the software Cutadapt (http://cutadapt.readthedocs.org/en/stable/) subread, multiqc, edgeR, limma, limma-voom and deseq2 [44–47]. Once quality was assessed, data were mapped to the human GRCh38 build reference sequence (iRbase release 225). Next, the aligned reads were obtained with miRbase v22. Alignments and quantification were performed using Subread and Rsubread Packages [47, 48].

A multi-dimensional scaling (MDS) plot was used to analyse sample distribution according to miRs expression values. miRs with very low counts across all libraries provide little evidence for differential expression. Consequently, these miRs were filtered out prior to further analysis. Subsequently, trimmed mean of M-values (TMM) normalization method was used to eliminate composition biases between libraries [49]. Specific dispersions per gene with a negative binomial distribution were also estimated [50, 51].

### Prediction of miRNA targets and over-representation analysis

Four packages (edgeR, voom, limma-voom and deseq2) were used to analyse differential expression of miRs between patient and control samples. Functional analysis of target genes for statistically different miRs was performed by two different methodologies: ORA (over-representation analysis, FDR < 0.05) and GSEA (Gene set enrichment analysis) implemented in clusterProfiler of R [52]. Data analysed by GSEA was arranged by miRs Fold Change log values. The enrichment analysis was performed to obtain the significant KEGG pathways or GO terms in both methodologies.

### qPCR validation of differentially expressed miRNAs

Reverse transcription reactions were performed using the TaqMan miR Reverse Transcription kit, miR-specific stem-loop primers (Part No. 4366597, Applied Biosystems. Inc, Foster City, CA; USA) and 100 ng of cell-free RNA in a 20 µL RT reaction. Real-time PCR reactions were performed in triplicate, using 5 µL TaqMan 2 × Universal PCR Master Mix (Applied Biosystems. Inc, Foster City, CA; USA) with No UNG, 0.5 µL TaqMan Small RNA assay (20x) (Applied Biosystems. Inc, Foster City, CA; USA), 3.5 µL of nuclease free water and 1 µL of RT product. Real-time PCR was carried out on an Applied BioSystems QuantStudio 5 (Applied Biosystems. Inc, Waltham, CA; USA) programmed as follows: 50 °C for 2 min, 95 °C for 10 min followed by 45 cycles of 95 °C for 15 s and 60 °C for 1 min. Selected miRs were validated using TaqMan™ MicroRNA Assay (Applied Biosystems): hsa-miR-19b-3p (Assay ID: 00396), hsa-miR-192-5p (Assay ID 000491), hsa-miR-322b-3p (Assay ID: 244080_mat), hsa-miR-122-5p (Assay ID: 002245), hsa-miR-206 (Assay ID: 000510). SmallRNA-seq data allowed the identification of miRs with similar and constant counts among samples. hsa-miR-16-5p and hsa-miR-191-5p were found to be feasible miRs for data normalization. hsa-miR-191-5p (Assay ID 002299), one of the most stable miRs in terms of read counts, previously used as an endogenous control [53, 54] was selected for data normalization in plasma samples.

Fold-change data were obtained using the delta-delta CT method (*2-ΔΔCT*) [55].

### Statistical analysis

Mann–Whitney U tests was used to compare miR fold-change values as a continuous variable in the different groups (healthy controls and LGMD, DMD or FSHD patients). The miR diagnostic test was validated by ROC curves analysis: area under the curve (AUC), diagnostic sensitivity and specificity, positive and negative predictive values. Optimal cut-off points were determined by highest sensitivity plus specificity and efficiency values. *P*-values: **** *p*-value < 0.0001, ***p-value < 0.001, **p-value < 0.01 or *p-value < 0.05 were considered statistically significant.

A series of Spearman’s Rho correlation analyses among clinical parameters and validated miRs were performed. A correlation result in which a Rs value was found between 0.8 to 1 indicates a strong correlation between the pair of groups tested. Statistical analysis and correlations were performed using Graphpad Prism 9.0 (GraphPad Software, San Diego, CA, USA).

## Supplementary Information

**Additional file 1: Table S1**: Clinical and biochemical features of DMD patients.

**Additional file 2: Table S2**: Clinical and biochemical features of FSHD patients.

**Additional file 3: Table S3**: Molecular signature of circulating miRs differentiating LGMD from other neuromuscular dystrophies.

**Additional file 4: Table S4**: Spearman coeficients among differentially expressed miRs and clinical or biochemical parameters in LGMD patients.

**Additional file 5: Fig. S1**: Receiver operating curves (ROC) of circulating miRNAs separate DMD and FSHD patients from healthy controls. (A) ROC curves were represented for miR-19b-3p, miR-122-5p, miR-192-5p, miR-206-5p and miR-323b-3p using the whole cohort of healthy participants (Controls) (n = 5) and DMD patients (n =5). (B) ROC curves were represented for miR-19b-3p, miR-122-5p, miR-192-5p, and miR-206-5p using the whole cohort of healthy participants (Controls) (n = 4) and FSHD patients (n =4). AUC, % CI, cut-off and P-values for indicated miRs are included in their respective ROC curves.

## Data Availability

Raw smallRNA-seq dataset obtained in this study (including LGMD and control samples) were deposited on Gene Expression Omnibus (GEO) repository (GSE208206, https://www.ncbi.nlm.nih.gov/geo/).

## Abbreviations

ALP: Alkaline phosphatase
AUC: Area under de curve
CI: Confidence interval
CK: Creatine kinase
DMD: Duchenne muscular dystrophy
FSHD: Facioscapulohumeral muscular dystrophy
FSH: Follicle-stimulating hormone
FSS: Functional System Score
LGMD: Limb girdle muscular dystrophy
LH: Luteinizing hormone
MDS: Multi-dimensional scaling
PG: Progesterone
PINP: Procollagen 1 intact N-terminal propeptide
PTH: Parathyroid hormone
ROC: Receiver operating characteristic curve
TMM: Trimmed mean of M-values
