# Supplementary material for: Identification of circulating miRNAs differentially expressed in patients with Limb-girdle, Duchenne or facioscapulohumeral muscular dystrophies
Source: Orphanet J Rare Dis. 2022 Dec 27;17:450. doi: 10.1186/s13023-022-02603-3 (PMC9793535; doi:10.1186/s13023-022-02603-3)
Supplement: Supplementary file 1 — Additional file 1: Table S1: Clinical and biochemical features of DMD patients. [file 13023_2022_2603_MOESM1_ESM.docx]

**Supplementary Table 1: Clinical and biochemical features of DMD patients**

| **Patient #** | DMD 4.1 | DMD 9.1 | DMD 12.1 | DMD 15.1 | DMD 17.1 |
| --- | --- | --- | --- | --- | --- |
| **Gene mutation** | Del exons 3-6 | NM_004006.3(DMD): c.103C>T (p.Gln35Ter) | Del exons 47 & 48 | NM_004006.2(DMD):exon 3 Duplication, c.116dupA; (p.Asn39Lysfs*5 ) | NM_004006.2(DMD):exon 28 Nonsense, c.3862A>T  (p.Lys1288*) |
| **Sex** | M | M | M | M | M |
| **Early/Late onset , Age** | Early, 14 | Early, 11 | Early, 2 | Early, 3 | Early, 13 |
| **Age last evaluation** | 24 | 18 | 23 | 19 | 22 |
| **Limb weakness ^a^** | 1 | 1 | 3 | 1 | 3 |
| **WCB^b^, Age** | YES, 10 | NO | YES, 10 | YES, 11 | YES, 13 |
| **Functional System Score (FSS)^c^** | 2 | 2 | 3 | 3 | 3 |
| **Cardiopathy** | NO | NO | YES | YES | YES |
| **Non-invasive ventilation required, Age** | NO | NO | YES, 21 | YES, 21 | YES, 15 |
| **Muscle biopsy** | NO | NO | NO | YES | YES |
| **Calcium (mg/dL)** | 9,2 | 9,7 | 9,0 | 9,7 | 9,5 |
| **iP (mg/dL)** | 3,6 | 4,6 | 3,4 | 3,8 | 4,4 |
| **Vit D3 (ng/mL)** | 18,8 | 21,3 | 34,3 | 24 | 8,8 |
| **PINP (ng/mL)** | 21,8 | 460 | 53,7 | 97 | 58,7 |
| **ALP (IU/L)** | 59 | 116 | 70 | 74 | 51 |
| **PTH (pg/mL)** | 54,4 | 20,7 | 66 | 29,2 | 23,1 |
| **PG (ng/mL)** | 0,25 | 0,31 | 0,27 | 2,45 | 2,59 |
| **FSH (mU/mL)** | 3,5 | 2,3 | 2 | 1,9 | 11,1 |
| **LH (mU/mL)** | 3,2 | 2,8 | 7,4 | 8,7 | 5 |
| **CK (IU/L)** | 3230,25 | 12300 | 693 | 1618 | 1342 |

^a^ 1: Proximal; 2: Distal; 3: Both Proximal & Distal

^b^ WCB: wheel-chair bound

^c^ 1: Fast/Severe; 2: moderate/progressive; 3: mild/long-term
