# Supplementary material for: Identification of circulating miRNAs differentially expressed in patients with Limb-girdle, Duchenne or facioscapulohumeral muscular dystrophies
Source: Orphanet J Rare Dis. 2022 Dec 27;17:450. doi: 10.1186/s13023-022-02603-3 (PMC9793535; doi:10.1186/s13023-022-02603-3)
Supplement: Supplementary file 2 — Additional file 2: Table S2: Clinical and biochemical features of FSHD patients. [file 13023_2022_2603_MOESM2_ESM.docx]

**Supplementary Table 2: Clinical and biochemical features of FSHD patients**

| **Patient #** | FSHD 2.1 | FSHD 3.1 | FSHD 11.1 | FSHD 13.1 |
| --- | --- | --- | --- | --- |
| **Muscular Distrophy** | FSHD1 | FSHD1 | FSHD1 | FSHD1 |
| **D4Z4 Reduced Allele (DRA) size** | 17 Kb | 24 Kb | 18 Kb | 17 Kb |
| **Sex** | F | F | F | F |
| **Early/Late onset , Age** | Early, 7 | Late, 35 | Late, 23 | Early, 21 |
| **Age last evaluation** | 50 | 47 | 23 | 29 |
| **Limb weakness ^a^** | 3 | 2 | 2 | 1 |
| **WCB^b^, Age** | YES/50 | NO | NO | NO |
| **Functional System Score (FSS)^c^** | 3 | 1 | 1 | 1 |
| **Cardiopathy** | NO | NO | NO | NO |
| **Non-invasive ventilation required** | NO | NO | NO | NO |
| **Muscle biopsy** | NO | YES | NO | YES |
| **Calcium (mg/dL)** | 9,1 | 9,2 | 9,0 | 9,2 |
| **iP(mg/dL)** | 4,2 | 2,9 | 3,5 | 4,5 |
| **Vit D3 (ng/mL)** | 7,4 | 28,1 | 42,8 | 21,4 |
| **PINP (ng/mL)** | 109 | 91,3 | 38,4 | 30,5 |
| **ALP (IU/L)** | 85 | 102 | 49 | 99 |
| **PTH (pg/mL)** | 50,4 | 56,8 | 34 | 47,3 |
| **PG (ng/mL)** | 0,6 | 1,6 | 0,16 | 0,08 |
| **FSH (mU/mL)** | 32,9 | 15,9 | 4,5 | 2,3 |
| **LH (mU/mL)** | 48,1 | 17 | 3,9 | 3,8 |
| **CK (IU/L)** | 237 | 385 | 96 | 108 |

^a^ 1: Proximal; 2: Distal; 3: Both Proximal & Distal

^b^ WCB: wheel-chair bound

^c^ 1: Fast/Severe; 2: moderate/progressive; 3: mild/long-term
