# Supplementary material for: Identification of circulating miRNAs differentially expressed in patients with Limb-girdle, Duchenne or facioscapulohumeral muscular dystrophies
Source: Orphanet J Rare Dis. 2022 Dec 27;17:450. doi: 10.1186/s13023-022-02603-3 (PMC9793535; doi:10.1186/s13023-022-02603-3)
Supplement: Supplementary file 3 — Additional file 3: Table S3: Molecular signature of circulating miRs differentiating LGMD from other neuromuscular dystrophies. [file 13023_2022_2603_MOESM3_ESM.docx]

**Supplementary Table 3: Molecular signature of circulating miRs differentiating LGMD from other neuromuscular dystrophies**

| **Differentially expressed miR** | **miR-122-5p** | **miR-192-5p** | **miR-323b-3p** |
| --- | --- | --- | --- |
| **LGMD** | Up (II) | Up (II) | Down (I) |
| **DMD** | Up (II) | Up (I) | Up (I) |
| **FSHD** | Up (I) | Up (II) | - |

(**-**): No difference vs healthy controls

(**I**): Difference ≥2-10 fold vs healthy matched controls

(**II**): Difference ≥10-60 fold vs healthy matched controls
