# Supplementary material for: Identification of circulating miRNAs differentially expressed in patients with Limb-girdle, Duchenne or facioscapulohumeral muscular dystrophies
Source: Orphanet J Rare Dis. 2022 Dec 27;17:450. doi: 10.1186/s13023-022-02603-3 (PMC9793535; doi:10.1186/s13023-022-02603-3)
Supplement: Supplementary file 4 — Additional file 4: Table S4: Spearman coeficients among differentially expressed miRs and clinical or biochemical parameters in LGMD patients. [file 13023_2022_2603_MOESM4_ESM.docx]

**Supplementary Table 4: Spearman coeficients among differentially expressed miRs and clinical or biochemical parameters in LGMD patients**

|  | **2^-ΔΔCt^ Hsa-miR-19b-3p** | | **2^-ΔΔCt^ Hsa-miR-122-5p** | | **2^-ΔΔCt^ Hsa-miR-192-5p** | | **2^-ΔΔCt^ Hsa-miR-206-5p** | | **2^-ΔΔCt^ Hsa-miR-323b-3p** | |
| --- | --- | --- | --- | --- | --- | --- | --- | --- | --- | --- |
|  | **Spearman R** | **P-value** | **Spearman R** | **P-value** | **Spearman R** | **P-value** | **Spearman R** | **P-value** | **Spearman R** | **P-value** |
| **BMI (kg/m^2^)** | 0.714 | 0.088 | -0.286 | 0.556 | -0.321 | 0.498 | -0.314 | 0.564 | -0.286 | 0.556 |
| **ALP (IU/L)** | **-0.928** | **0.017** | -0.580 | 0.233 | **-0.841** | **0.044** | -0.400 | 0.517 | 0.058 | 0.933 |
| **FSH (mU/mL)** | 0.036 | 0.936 | -0.464 | 0.302 | -0.750 | 0.066 | -0.143 | 0.803 | 0.071 | 0.906 |
| **LH (mU/mL)** | 0.464 | 0.302 | -0.250 | 0.595 | -0.464 | 0.302 | -0.771 | 0.103 | 0.170 | 0.840 |
| **PG (ng/mL)** | -0.464 | 0.302 | 0.143 | 0.783 | 0.179 | 0.713 | 0.600 | 0.242 | -0.107 | 0.840 |
| **Vit D3 (ng/mL)** | -0.429 | 0.354 | -0.750 | 0.066 | **-0.893** | **0.012** | -0.657 | 0.175 | -0.179 | 0.713 |
| **PTH (pg/mL)** | **0.821** | **0.034** | 0.143 | 0.783 | 0.071 | 0.906 | -0.086 | 0.919 | 0.000 | 1.000 |
| **PINP (ng/mL)** | -0.771 | 0.103 | -0.543 | 0.297 | -0.829 | 0.058 | -0.400 | 0.517 | 0.143 | 0.803 |
| **CK (IU/L)** | 0.571 | 0.200 | **0.929** | **0.007** | **0.857** | **0.024** | 0.429 | 0.419 | 0.536 | 0.236 |
| **FSS** | 0.060 | 0.914 | -0.120 | 0.800 | 0.199 | 0.667 | -0.494 | 0.367 | -0.777 | 0.236 |
