# Supplementary figures and images for: Identification of circulating miRNAs differentially expressed in patients with Limb-girdle, Duchenne or facioscapulohumeral muscular dystrophies
Source: Orphanet J Rare Dis. 2022 Dec 27;17:450. doi: 10.1186/s13023-022-02603-3 (PMC9793535; doi:10.1186/s13023-022-02603-3)

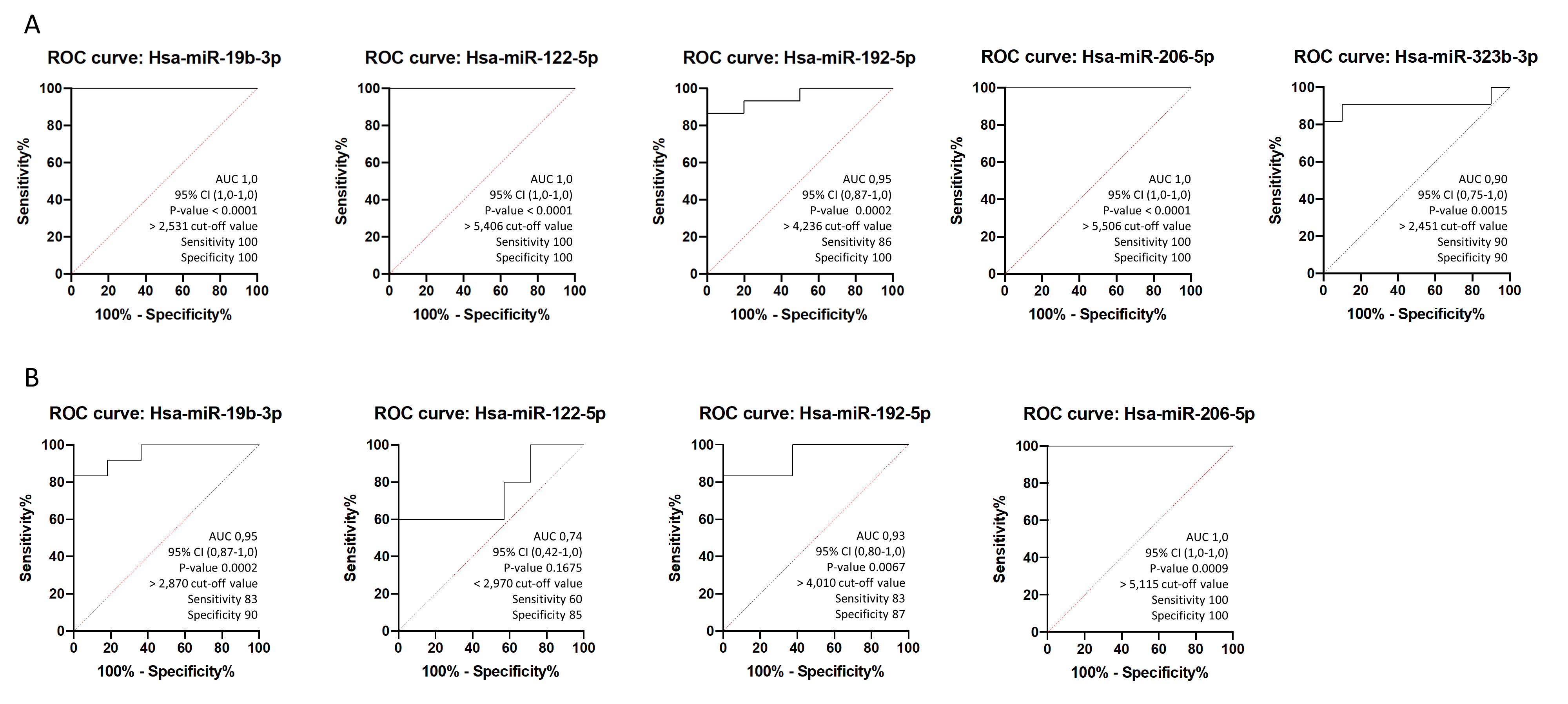

Supplement: Supplementary file 5 — Additional file 5: Fig. S1: Receiver operating curves (ROC) of circulating miRNAs separate DMD and FSHD patients from healthy controls. (A) ROC curves were represented for miR-19b-3p, miR-122-5p, miR-192-5p, miR-206-5p and miR-323b-3p using the whole cohort of healthy participants (Controls) (n = 5) and DMD patients (n =5). (B) ROC curves were represented for miR-19b-3p, miR-122-5p, miR-192-5p, and miR-206-5p using the whole cohort of healthy participants (Controls) (n = 4) and FSHD patients (n =4). AUC, % CI, cut-off and P-values for indicated miRs are included in their respective ROC curves. [file 13023_2022_2603_MOESM5_ESM.tif]
